# Serum C-reactive protein increases the risk of venous thromboembolism: a prospective study and meta-analysis of published prospective evidence

**DOI:** 10.1007/s10654-017-0277-4

**Published:** 2017-07-17

**Authors:** Setor K. Kunutsor, Samuel Seidu, Ashley W. Blom, Kamlesh Khunti, Jari A. Laukkanen

**Affiliations:** 1 School of Clinical Sciences, University of Bristol, Learning and Research Building (Level 1), Southmead Hospital, Southmead Road, Bristol, BS10 5NB UK; 2 Leicester Diabetes Centre, Leicester General Hospital, Gwendolen Road, Leicester, LE5 4WP UK; 3 Diabetes Research Centre, University of Leicester, Leicester General Hospital, Gwendolen Road, Leicester, LE5 4WP UK; 4 Department of Medicine, Institute of Public Health and Clinical Nutrition, University of Eastern Finland, Kuopio, Finland; 5 Department of Medicine, Central Finland Central Hospital, Jyväskylä, Finland

**Keywords:** C-reactive protein, Cohort study, Venous thromboembolism, Deep vein thrombosis, Pulmonary embolism

## Abstract

Evolving debate suggests that C-reactive protein (CRP) might be associated with the development of venous thromboembolism (VTE); however, the evidence is conflicting. We aimed to assess the prospective association of CRP with VTE risk. C-reactive protein was measured in serum samples at baseline from 2420 men aged 42–61 years, from the Kuopio Ischemic Heart Disease study. Within-person variability in CRP levels was corrected for using repeat measurements of CRP taken 11 years after baseline. Incident VTE events (n = 119) were recorded during a median follow-up of 24.7 years. The age-adjusted regression dilution ratio for log_e_ CRP was 0.57 [95% confidence interval (CIs): 0.51–0.64]. In age-adjusted Cox regression analysis, the hazard ratio (95% CIs) for VTE per 1 standard deviation (SD) increase in log_e_ baseline CRP was 1.17 (0.98–1.40). Further adjustment for several established and emerging risk factors did not alter the association. In a meta-analysis of nine population-based studies (including the current study) comprising 81,625 participants and 2225 VTE cases, the fully-adjusted risk estimate for VTE was 1.14 (1.08–1.19) per SD increase in log_e_ baseline CRP. In a pooled dose–response analysis, a linear association between CRP and VTE risk was suggested (*P* for nonlinearity = 0.272). The pooled risk estimate for VTE per 5 mg/l increment in CRP levels was 1.23 (1.09–1.38). C-reactive protein was only modestly associated with VTE risk in the primary analysis. Pooled evidence, however, suggests that elevated CRP is associated with greater VTE risk, consistent with a linear dose–response relationship.

**Electronic supplementary material:**

The online version of this article (doi:10.1007/s10654-017-0277-4) contains supplementary material, which is available to authorized users.

## Introduction

Venous thromboembolism (VTE), which comprises of deep vein thrombosis (DVT) and pulmonary embolism (PE), affects several millions of people and is an important cause of morbidity, mortality and increasing health care costs globally [1]. Despite advances in treatment options, VTE remains a global public health problem [2] and there has been no decrease in its incidence for the past several decades. Anticoagulation, which is the mainstay of VTE treatment, is commonly associated with an increased risk of major bleeding and subsequently high case fatality rates [3]. Major risk factors for VTE include immobilisation, trauma, surgery, hormonal therapy, obesity, and disorders of hypercoagulation [4]. However, in a substantial number of VTE cases, the causes are unknown [5]. It therefore appears that there may be several unknown factors involved in the pathogenesis of VTE. Identification of risk factors of VTE which have predictive or causal relevance could aid in the development of preventive strategies, especially in high risk individuals.

C-reactive protein (CRP), a non-specific marker for inflammation which is produced by the liver, is known to play a central role in the aetiopathogenesis of arterial atherothrombosis [6]. Cardiovascular disease (CVD) and VTE are closely linked [7–9] and share common antecedent risk factors [10], and there is an evolving debate that inflammation or CRP might also be linked to the development of VTE [11, 12]; however, the emerging evidence is controversial. A number of population-based prospective cohort studies have reported on the associations of circulating levels of CRP with subsequent risk of VTE, but the results have been conflicting. Whereas majority of studies have shown no evidence of any associations [13–16], only a few studies have reported some evidence of an association between CRP and VTE risk [17–19]. Given that CRP is known to exert prothrombotic effects and is closely linked with several risk factors for VTE [20–22], we hypothesized that increased baseline circulating levels of CRP will be associated with subsequent risk of VTE. In this context, we aimed to evaluate the nature and magnitude of the prospective association of CRP with risk of VTE in a population-based cohort of 2420 men with no previous history of VTE from eastern Finland.

Repeat measurements of CRP performed several years apart in a random sample of participants enabled quantification of within-person variability in CRP levels. Finally, with the availability of a number of published articles that have evaluated the prospective association between CRP and VTE, this offered the opportunity to perform a systematic review and meta-analysis, thereby re-evaluating the nature and magnitude of the association in a larger representative sample of participants and VTE cases.

## Methods

We conducted this study in accordance with STROBE (STrengthening the Reporting of OBservational studies in Epidemiology) guidelines for reporting observational studies in epidemiology (Appendix 1).

### Study design and participants

Participants included in this analysis were part of the Kuopio Ischemic Heart Disease (KIHD) study, a single-centre, population-based prospective cohort study, which was set up to evaluate risk factors for CVD and other chronic disease outcomes. The study design and recruitment methods have been described previously [23, 24]. A representative sample of men aged 42–61 years who were inhabitants of the city of Kuo-pio and its surrounding rural communities in eastern Finland were invited for baseline examinations carried out between March 1984 and December 1989. A total of 3433 potentially eligible men were randomly selected and of this number, 3235 were found to be eligible. Among the eligible men, 2682 (78%) provided consent to participate in the study; 186 did not respond to the invitation and 367 declined to participate in the research study. In total, 2420 men who had complete information on CRP, relevant covariates, and VTE outcomes were included in the final analyses. All study procedures were approved by the Research Ethics Committee of the University of Eastern Finland and were conducted according to the Declaration of Helsinki. Written informed consent was provided by each participant.

### Assessment of risk markers

Baseline information was collected by physical examination, blood samples, and self-administered questionnaires. Blood pressure was recorded by an experienced nurse with a random-zero sphygmomanometer (Hawskley, UK) after 5 and 10 min of rest in a seated position [25]. Body mass index (BMI) was estimated as weight in kilograms divided by the square of height in meters. Blood samples were taken after 8 and 10 a.m. after an overnight fast. In addition, participants were to abstain from alcohol consumption for at least 3 days and smoking for at least 12 h prior to blood collection. Serum samples were stored frozen at −80 °C before measurements of lipids and biochemical analytes. Serum CRP measurements were made with an immunometric assay (Immulite High Sensitivity C-Reactive Protein Assay; DPC, Los Angeles, CA, USA), with repeat measurements performed in a random subset of participants at 11 years after the baseline measurements. The kinetic method (Thermo Fisher Scientific, Vantaa, Finland) was used to measure gamma-glutamyltransferase (GGT) activity. Fasting plasma glucose (FPG) was measured using the glucose dehydrogenase method (Merck, Darmstadt, Germany). For the assessments of age, smoking, alcohol consumption, baseline medical conditions, and medication history; participants completed self-administered health and lifestyle questionnaires [26]. The energy expenditure of physical activity was assessed using the validated KIHD 12-month leisure-time physical activity questionnaire [27, 28].

### Ascertainment of incident venous thromboembolism

All first lifetime VTE events that occurred from study entry to 2013 were included and were identified by searching the National Hospital Discharge Registry data by computer linkage and a comprehensive review of available hospital records, wards of health centres, health practitioner questionnaires, death certificate registers, autopsy registers, and medico-legal reports. No losses to follow-up were recorded as all participants in the KIHD study (using Finnish personal identification codes) are under continuous surveillance for the development of new outcomes including VTE cases. Documents were cross-checked in detail and VTE events were validated by two physicians. The diagnosis of DVT or PE required positive imaging tests.

### Statistical analyses

#### Prospective cohort analyses

Skewed variables (CRP, triglycerides, and GGT) were log transformed to achieve approximately normal distributions. Descriptive analyses were conducted to summarize baseline characteristics of participants, with means (SD) or medians (IQR, interquartile range) reported for continuous variables and percentages for categorical variables. Time-to-event analyses were conducted using Cox proportional hazard regression models after confirming assumptions of proportionality of hazards using Schoenfeld residuals [29]. To quantify and correct for within-person variability in CRP levels, which is, the extent to which an individual’s CRP measurements vary around the long-term average exposure levels (“usual levels”) [30], adjusted regression dilution ratios (RDRs) were estimated by regressing available repeat measurements on baseline values [31]. The RDR assumes that the “usual levels” of CRP represents the true long-term exposure of CRP levels on VTE risk. To characterize the shape of the association between CRP and VTE risk, hazard ratios (HRs) were calculated within quartiles of baseline CRP levels and plotted against mean CRP levels within each quartile using floating absolute risks [26]. C-reactive protein was modeled continuously [per 1 standard deviation (SD) higher log_e_ CRP levels] and as categories (quartiles) defined according to the baseline distribution of CRP levels. The SD of baseline log_e_ CRP was 0.97 (equivalent to approximately three-fold higher circulating CRP, as e^0.97^ = 2.64). The HRs were adjusted progressively for (1) age; and (2) other established risk factors and potential confounders [BMI, systolic blood pressure (SBP), history of hypertension, prevalent coronary heart disease (CHD), smoking status, history of diabetes, total cholesterol, lipid medication, physical activity, and GGT]. These confounders were selected based on their potential as confounders as a result of their known associations with VTE outcomes and observed associations with CRP using the available data [32] or evidence from previous research. Formal tests of interaction were used to assess statistical evidence of effect modification by categories of pre-specified clinically relevant individual level characteristics.

#### Systematic review and meta-analysis

We conducted a meta-analysis of published studies reporting on the association between CRP and risk of VTE, using a predefined protocol and reported in accordance with PRISMA and MOOSE guidelines [33, 34] (Appendices 2 and 3). Published observational population-based prospective (cohort, case cohort, or nested case–control) studies with at least one year of follow-up that evaluated the associations between baseline levels of CRP and risk of VTE up to January 2017, were sought using computer-based databases (MEDLINE, EMBASE, and Web of Science). The computer-based searches combined free and MeSH search terms and combined key words related to the exposure (e.g., “C-reactive protein”) and outcome (e.g., “venous thromboembolism”, “deep vein thrombosis”, “pulmonary embolism”). We placed no restrictions on language or the publication date. Details of the search strategy are reported in Appendix 4. Two independent authors (S.K.K., S.S.) performed screening, data extraction, and quality assessments. Discrepancies were discussed and agreement reached by adjudication of a third author (J.A.L.). Information was extracted on study characteristics such as study design, publication year, geographical location, baseline age, duration of follow-up, sample size and number of VTE events, and risk estimates for the most adjusted models. We assessed study quality using the nine-star Newcastle–Ottawa Scale (NOS) [35] as described previously [36]. Summary measures were presented as relative risks (RRs) with 95% confidence intervals (CIs). Following Cornfield’s rare disease assumption [37], hazard ratios and odds ratios were assumed to approximate the same measure of RR. To enable a consistent approach to the meta-analysis and enhance comparison with the primary analysis, reported study-specific risk estimates were also transformed to per SD increase in or as extreme quartiles of CRP using standard statistical methods, [38, 39] which have been described in detail previously [40, 41] and in Appendix 5. The associations of “usual levels” of CRP with VTE risk were estimated using the RDR derived from the KIHD Study. Summary RRs were pooled using a random effects model to minimize the effect of between-study heterogeneity [42]. Subsidiary analysis used fixed effects models. Statistical heterogeneity between studies was quantified using standard Chi square tests and the I^2^ statistic [43]. We also assessed the potential for small study effects such as publication bias through formal tests, namely Begg’s funnel plots [44] and Egger’s regression symmetry test [45].

We also performed a pooled dose–response association of circulating levels of CRP with risk of VTE using data from published studies. A 2-step generalized least-squares trend estimation (GLST) analysis as described by Greenland and Orsini [39, 46], was used to compute study-specific slopes (linear trends) from the correlated natural logs of the RRs across categories of CRP. Potential nonlinear dose–response relationships were examined by modeling levels of CRP using restricted cubic splines. Details of the methodology have been described in previous reports [41, 47]. Briefly, this method requires that the number of cases, person-years of follow-up or non-cases, and the RRs with the variance estimates for at least three quantitative categories of CRP levels are known. The median or mean level of CRP for each category was assigned to each corresponding RR. If data were not available, we estimated the median using the midpoint of each category. When the highest or lowest category was open, we assumed it to be the same amplitude as the adjacent category. All statistical analyses were conducted using Stata version 14 (Stata Corp, College Station, Texas).

## Results

### Baseline characteristics

Table 1 and Appendix 6 summarize the baseline characteristics of the 2420 participants included in the present analysis according to quartiles of CRP and development of VTE events during follow-up respectively. The mean age of study participants was 53 (SD, 5) years. The mean (SD) log_e_ CRP level was 0.34 (0.97) mg/l. There were no evident differences in baseline characteristics between VTE cases and participants who did not develop VTE during follow-up, except for smoking status. There was a higher proportion of smokers in the group who did not develop VTE during follow-up compared with the group that developed VTE during follow-up.

**Table 1:** Baseline participant characteristics by quartiles of CRP

	Overall (N = 2420) mean (SD) or median (IQR) or n (%)	Quartile 1 mean (SD) or median (IQR) or n (%)	Quartile 2 mean (SD) median (IQR) or n (%)	Quartile 2 mean (SD) or median (IQR) or n (%)	Quartile 2 mean (SD) or median (IQR) or n (%)
CRP (mg/l)	1.30 (0.71–2.49)	0.49 (0.36–0.60)	0.96 (0.83–1.13)	1.76 (1.53–2.09)	4.4 (3.21–6.97)
*Questionnaire/prevalent conditions*					
Age at survey (years)	53.2 (5.0)	52.6 (5.3)	53.2 (4.9)	53.5 (4.8)	53.6 (4.9)
Alcohol consumption (g/week)	75.6 (136.5)	58.7 (104.0)	66.2 (117.2)	78.6 (137.1)	98.9 (174.2)
History of diabetes	99 (4.1)	11 (1.8)	15 (2.5)	29 (4.8)	44 (7.3)
Current smokers	766 (31.7)	132 (21.8)	146 (24.2)	201 (33.1)	287 (47.6)
History of hypertension	736 (30.4)	134 (22.1)	191 (31.6)	186 (30.6)	225 (37.3)
History of CHD	620 (25.6)	103 (17.0)	139 (23.0)	159 (26.2)	219 (36.3)
Lipid medication	16 (0.7)	3 (0.5)	1 (0.2)	5 (0.8)	7 (1.2)
*Physical measurements*					
BMI (kg/m^2^)	26.9 (3.6)	25.4 (2.9)	26.6 (3.0)	27.4 (3.5)	28.2 (4.1)
SBP (mmHg)	134 (17)	131 (15)	134 (17)	136 (18)	137 (18)
DBP (mmHg)	89 (11)	87 (10)	89 (10)	89 (11)	90 (11)
Physical activity (kj/day)	1546 (1482)	1517 (1233)	1573 (1724)	1524 (1425)	1568 (1506)
*Lipid markers*					
Total cholesterol (mmol/l)	5.92 (1.09)	5.76 (1.04)	5.93 (1.07)	6.01 (1.08)	5.98 (1.16)
HDL-C (mmol/l)	1.30 (0.30)	1.39 (0.33)	1.30 (0.30)	1.30 (0.28)	1.21 (0.27)
Triglycerides (mmol/l)	1.10 (0.80–1.56)	0.92 (0.70–1.27)	1.08 (0.81–1.55)	1.17 (0.85–1.61)	1.25 (0.91–1.81)
*Metabolic, renal, and inflammatory markers*					
Fasting plasma glucose (mmol/l)	5.36 (1.28)	5.12 (0.64)	5.27 (1.02)	5.42 (1.35)	5.66 (1.77)
Serum creatinine (µmol/1)	89.6 (20.7)	89.7 (11.9)	89.7 (12.9)	89.0 (13.1)	90.0 (35.3)
GGT (U/L)	20 (15–33)	16 (12–24)	19 (14–28)	22 (16–37)	26 (18–43)

*BMI* body mass index, *CHD* coronary heart disease, *CRP*, C-reactive protein, *DBP* diastolic blood pressure, *GGT* gamma-glutamyltransferase, *HDL-C* high-density lipoprotein cholesterol, *SD* standard deviation, *SBP* systolic blood pressure, *VTE* venous thromboembolism

### Correction for within-person variability in CRP levels

In a random subset of 744 participants, repeat measurements of CRP were taken at 11 years after the baseline measurements during the follow-up period. Overall, the age-adjusted RDR of log_e_ CRP was 0.57 (95% CI 0.51–0.64), which suggests that the association of CRP with VTE risk using baseline measurements of CRP could under-estimate the association by [(1/0.57)−1] × 100 = 75%.

### C-reactive protein and risk of venous thromboembolism

#### Prospective cohort analysis

**Fig. 1:**
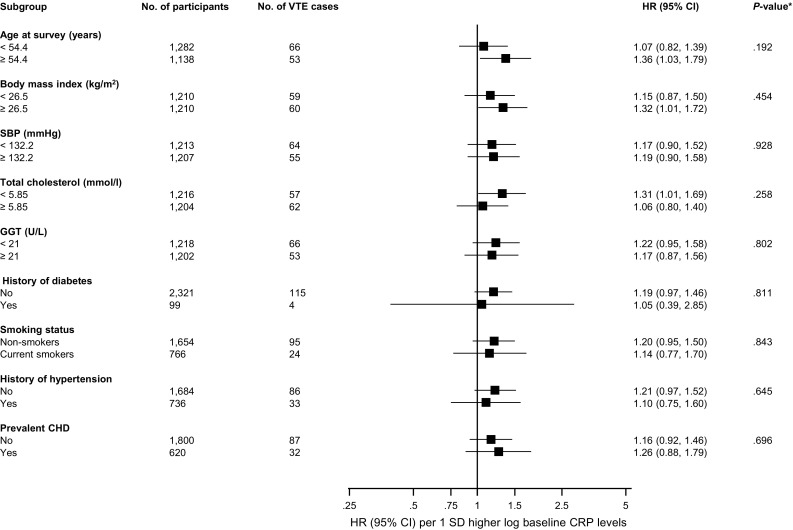
Hazard ratios for baseline values of C-reactive protein and venous thromboembolism risk by several participant level characteristics. Hazard ratios are adjusted for age, body mass index, SBP, history of hypertension, prevalent CHD, smoking status, history of diabetes, total cholesterol, lipid medication, physical activity, and GGT; CHD, coronary heart disease; CI, confidence interval; CRP, C-reactive protein; GGT, gamma-glutamyltransferase; HR, hazard ratio; SD, standard deviation; SBP, systolic blood pressure; VTE, venous thromboembolism; *, *P* value for interaction; cut-offs used for age, body mass index, SBP, total cholesterol, and GGT are median values

During a median follow-up of 24.7 (interquartile range 17.1–27.1) years, 119 VTE events (annual rate 2.29/1000 person-years at risk; 95% CI 1.92–2.75) were ascertained. A trend towards a log-linear association was observed between CRP and risk of VTE in both age- and multivariate-adjusted analysis (Appendix 7). The age-adjusted HR for VTE per 1 SD increase in log_e_ baseline CRP was 1.17 (95% CI 0.98–1.40) which remained unchanged 1.18 (95% CI 0.97–1.44) on further adjustment for several established risk factors and potential confounders (BMI, SBP, history of hypertension, prevalent CHD, smoking status, history of diabetes, total cholesterol, lipid medication, physical activity, and GGT). Alternatively, comparing the top versus bottom quartiles of CRP levels, the corresponding adjusted HRs were 1.39 (95% CI 0.84–2.30) and 1.38 (95% CI 0.80–2.39) respectively (Table 2). The corresponding adjusted HRs per 1 SD increase in usual log_e_ CRP levels were 1.32 (95% CI 0.96–1.80) and 1.34 (95% CI 0.95–1.90) respectively (Table 2). The association did not importantly vary across several clinical subgroups (*P* for interaction ≥ 0.10 for each; Fig. 1).

**Table 2:** Association of serum C-reactive protein and venous thromboembolism

Serum CRP (mg/l)	Events/total	Model 1		Model 2	
		HR (95% CI)	*P* value	HR (95% CI)	*P* value
Baseline CRP					
Per 1 SD increase	119/2420	1.17 (0.98–1.40)	0.089	1.18 (0.97–1.44)	0.098
Q1 (0.10–0.71)	29/606	ref		ref	
Q2 (0.72–1.29)	29/604	1.00 (0.60–1.67)	0.998	0.97 (0.58–1.63)	0.908
Q3 (1.30–2.49)	29/607	1.10 (0.66–1.85)	0.712	1.06 (0.62–1.82)	0.825
Q4 (≥2.50)	32/603	1.39 (0.84–2.30)	0.199	1.38 (0.80–2.39)	0.252
Usual CRP^a^					
Per 1 SD increase	119/2420	1.32 (0.96–1.80)	0.089	1.34 (0.95–1.90)	0.098
Q1 (0.10–0.71)	29/606	ref		ref	
Q2 (0.72–1.29)	29/604	1.00 (0.40–2.47)	0.998	0.95 (0.38–2.36)	0.908
Q3 (1.30–2.49)	29/607	1.19 (0.48–2.93)	0.712	1.11 (0.43–2.85)	0.825
Q4 (≥2.50)	32/603	1.79 (0.74–4.33)	0.199	1.76 (0.67–4.60)	0.252

Model 1: adjusted for age

Model 2: Model 1 plus body mass index, systolic blood pressure, history of hypertension, prevalent coronary heart disease, smoking status, history of diabetes, total cholesterol, lipid medication, physical activity, and gamma-glutamyltransferase

*CI* confidence interval, *CRP* C-reactive protein, *HR* hazard ratio, *ref* reference, *Q* quartile, *SD* standard deviation

^a^ indicates correction for within-person variability in values of CRP, that is, the extent to which an individual’s CRP measurements vary around a long-term average value (“usual CRP values”)

#### Meta-analysis of published studies

**Fig. 2:**
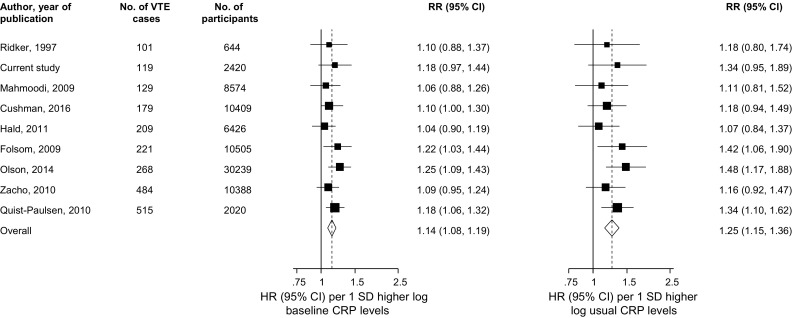
Prospective studies of C-reactive protein and risk of venous thromboembolism. The summary estimates presented were calculated using random effects models; relative risks are reported per 1 standard deviation (SD) increase in C-reactive protein levels; size of data markers are proportional to the inverse of the variance of the relative ratio; CI, confidence interval (*bars*); RR, relative risk; VTE, venous thromboembolism

**Fig. 3:**
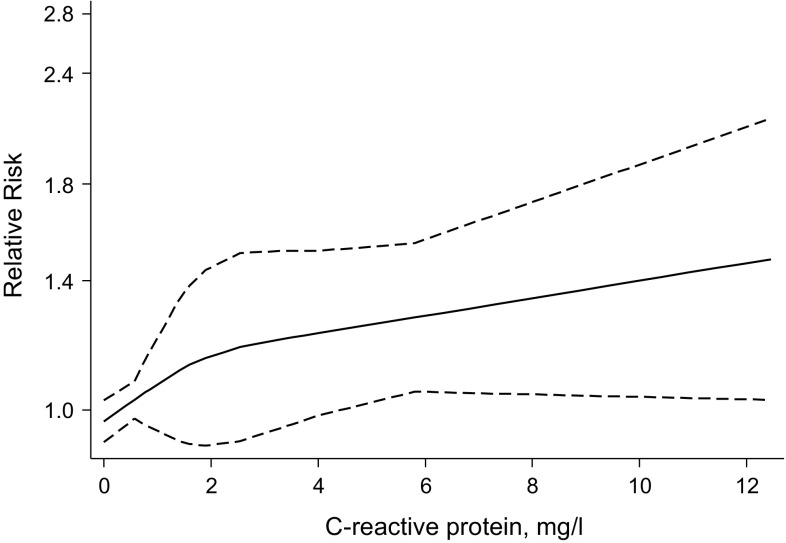
Dose-response relation between C-reactive protein and risk of venous thromboembolism for pooled results of studies providing relevant data. Adjusted relative risks and 95% confidence intervals (CIs *dashed lines*) are reported. Data were modeled with restricted cubic splines with 3 knots in random-effects dose–response models. The median value (0.30 mg/l) of the lowest reference range was used to estimate all relative risks. The vertical axes are on log scales. The following studies reported relevant data to model the dose–response relationship between C-reactive protein and venous thromboembolism risk [15–18]

We identified eight population-based prospective cohort studies reporting on the associations between circulating blood CRP and VTE risk (Appendices 8 and 9) [13–19, 48]. Including the current study, the pooled analysis comprised nine studies involving 81,625 participants and 2225 VTE cases. The pooled RRs for VTE per 1 SD higher baseline and usual CRP levels in fully-adjusted analyses were 1.14 (95% CI 1.08–1.19) and 1.25 (95% CI 1.15–1.36) respectively; (*I* ^*2*^ = 0%, 95% CI 0–65%; *P* = 0.648) (Fig. 2). The corresponding RRs were 1.36 (95% CI 1.20–1.54) and 1.72 (95% CI 1.38–2.13) respectively when comparing the top versus bottom quartiles of CRP levels. When a fixed effect model was employed, the summary RRs were identical to that of random-effects meta-analysis. There was no evidence of publication bias among contributing studies (Egger’s test *P* = 0.743) (Appendix 10). In pooled analysis of 4 studies providing relevant data [15–18], we found no evidence of statistically significant departure from linearity (*P* for nonlinearity = 0.272) between CRP levels and risk of VTE. Visual inspection of the plot was also consistent with an approximately linear shape (Fig. 3). The combined RR of VTE for a 5 mg/l increment in CRP level was 1.23 (95% CI 1.09–1.38).

### Comment

#### Summary of main findings

Our analysis of the population-based prospective study of middle-aged Finnish men provides no evidence of an association between elevated CRP levels and increased risk of developing VTE, though there was a trend towards a positive linear relationship. Though there was no evidence of effect modification across categories of several clinically relevant characteristics, the associations between CRP and VTE in older age and higher BMI groups were significant, which was not unexpected. The meta-analysis of nine prospective studies (including the current study) showed a positive association between CRP levels and incident VTE after controlling for several established risk factors and other potential confounders. Evidence was also lacking of heterogeneity and publication bias among contributing studies. Pooled estimates of studies with relevant data was consistent with a linear dose–response relationship to the association between CRP and VTE—characterised by a 23% increase in the risk of VTE for every 5 mg/l increment in circulating CRP levels.

### Comparison with previous studies

Though a number of prospective cohort studies have reported on the associations of circulating levels of CRP with subsequent risk of VTE, the majority of these studies have demonstrated no significant evidence of any association. The results of these previous studies are consistent with the findings of our primary analysis, which could be attributed to low power (low event rates) to detect an effect. Only a limited number of studies characterized by larger sample sizes (and comparatively higher event rates) have so far demonstrated significant evidence of an association between blood circulating CRP levels and VTE risk [17–19]. Our pooled analysis of all studies may have therefore provided enhanced power to show statistically significant evidence of an association between CRP levels and VTE risk. Except for one study [49], all previous studies did not correct for regression dilution bias; [50] which potentially results in the underestimation of the true association between an exposure and outcome, particularly for cohorts with long-term follow-up. It is thus possible that the inability of previous long-term follow-up cohort studies to demonstrate an association between CRP and VTE risk can be partly attributed to regression dilution [49, 51]. For example, in the Nord-Trøndelag Health Study (HUNT 2), a strong association was demonstrated between CRP and VTE risk for participants who experienced VTE within a year after blood sampling, whiles there was no evidence of an association in participants with more than 3 years between blood sampling and VTE events [17]. Regression dilution bias can either be addressed by conducting a time-varying analysis or correcting the risk estimates using the RDR. Indeed, the recent evaluation of the Tromsø study showed evidence of an association between repeated measurements of CRP and VTE risk (using time varying analysis) [49], compared to the previous analysis which employed a time-fixed analysis and showed no evidence of an association [15]. Our reproducibility substudies of CRP measurements within the KIHD study indicated a high within-person variability in CRP levels; which suggests that analyses using only single baseline measurements of CRP substantially underestimates the associations as we have shown.

#### Possible explanations for findings

An inflammatory hypothesis has been postulated in the pathogenesis of VTE, but the evidence is limited and unclear. Though inflammation as well as increased CRP levels are well known to increase the risk of atherothrombosis [13, 52] and may also promote hypercoagulable states, it is still uncertain if inflammation actually increases the risk of VTE. The overall findings from the current study lend some support to this inflammatory hypothesis. Indeed, several other inflammatory markers such as the interleukins and tumour necrosis factor alpha have been shown to be associated with VTE [53, 54]. Statins are known to decrease CRP levels (by up to 60%) independent of reductions in levels of low-density lipoprotein [55] and it has also been shown in both observational cohort as well as clinical intervention studies that statin treatment is associated with a reduction in risk of VTE [56, 57]. The beneficial effect of statins on VTE has been attributed to its anti-inflammatory effects [58]. Though statins also possess antithrombotic properties [59] which may explain the protective effect on VTE; it has also been postulated that the antithrombotic effects of statins are likely to be linked to their anti-inflammatory properties [60]. There have also been suggestions that atherosclerotic disease is an underlying condition and precedes the development of VTE [7]; however, evidence on the contrary suggests this is not the case [61]. The aetiopathogenic pathways underlying the association between CRP and VTE remain elusive, therefore mechanistic studies are needed to clarify the uncertainties. Collectively, the present study establishes an observational linear dose–response association to the link between CRP and VTE risk. Whether elevated CRP is a direct cause of VTE or just a risk marker remains unclear. However, Zacho and colleagues using a Mendelian randomization design have shown that genetically elevated CRP is not associated with VTE risk [16]. In another study, genetic polymorphisms that increase CRP levels were shown not to be associated with VTE risk [62]. Taking the evidence together, it can be argued that increased CRP is unlikely to have a causal relevance to VTE, but rather be a risk marker of VTE.

#### Implications of findings

C-reactive protein is an independent risk marker for CVD and may be of value in the discrimination and reclassification of individuals at risk for CVD [63]. Cardiovascular disease and VTE are closely linked conditions [7–9], share common risk factors [10], and may have common pathophysiological mechanisms; the current findings suggest that CRP may also play a role in the development of VTE. Large-scale studies are needed to replicate this association and investigate the potential relevance of CRP in VTE prevention. If CRP is demonstrated to have a role in preventing VTE, information on CRP levels may be of immense clinical benefit such as guiding the dosage and duration of anticoagulant therapy. Studies are also needed to establish if the protective effect of statin treatment on VTE risk is due to its CRP lowering effects.

#### Strengths and limitations

Our analysis had the advantage of utilizing a large-scale population-based prospective cohort design with selection of men who were nationally representative; involved a high response rate with no loss to follow-up; the long follow-up period of over 20 years; and the comprehensive analysis with adjustment for a broad panel of risk factors and potential confounders as well stratified analyses by several clinical relevant characteristics. Repeat measurements of CRP made within a random subset of individuals over time after baseline were available, which enabled correction for the extent of within-person variability in CRP over the long period of follow-up. Ideally, it would have been more appropriate to conduct a time-varying analysis as corrections using the RDR have been suggested to result in overcorrection of the risk estimates if the relationship between the exposure and outcome is not short term [64]. Indeed, it has recently been shown that corrections of the association between several atherosclerotic risk factors and VTE risk using RDRs consistently overestimated the risk estimates compared with time-varying analysis [51]. The same study showed that risk estimates for VTE based on baseline measurements (time-fixed analyses) corresponded well with those of time-varying analyses. We were unable to perform a time-varying analysis to allow for changes in CRP during follow-up, as we did not have the relevant data. The current risk estimate should therefore be interpreted with caution as there is a possibility that the true estimate lies between that of the time-fixed analysis without and with correction for regression dilution. Other strengths of the current study include the ability to conduct a pooled analysis of previous studies including the current study, which enhanced power to reliably assess the nature, magnitude, and shape of the association. In our pooled analysis, there was no evidence of heterogeneity or publication bias among contributing studies. There were limitations which deserve mention and include: (1) inability to generalize the findings to women and other population; (2) we had data on only total VTE which precluded the ability to conduct subgroup analyses of unprovoked or provoked VTE outcomes; (3) because of lack of appropriate data, we were unable to account for incident cancer as a time-varying covariate as this could partly account for the association between CRP and VTE; (4) inability to fully examine the impact of adjustment for potential confounders, because the review was based on variably adjusted data reported in the published literature; however, majority of included studies adjusted for major confounders; and (5) our dose–response analysis was limited to datapoints reported by only four studies.

## Conclusions

C-reactive protein was only modestly associated with VTE risk in this middle-aged Caucasian population, which may be attributed to the low event rate. Pooled evidence which enhanced statistical power, however, suggests that increased circulating CRP is associated with greater VTE risk and consistent with a linear dose–response relationship. Further research is needed to evaluate any potential relevance of CRP in VTE prevention.

## Electronic supplementary material

Below is the link to the electronic supplementary material.

Supplementary material 1 (DOC 575 kb)
