# Supplementary material for: Serum C-reactive protein increases the risk of venous thromboembolism: a prospective study and meta-analysis of published prospective evidence
Source: Eur J Epidemiol. 2017 Jul 17;32(8):657–67. doi: 10.1007/s10654-017-0277-4 (PMC5591345; doi:10.1007/s10654-017-0277-4)
Supplement: Supplementary file 1 — Supplementary material 1 (DOC 575 kb) [file 10654_2017_277_MOESM1_ESM.doc]

**SUPPLEMENTARY MATERIAL**

| **Appendix 1** | STROBE Statement |
| --- | --- |
| **Appendix 2** | PRISMA checklist |
| **Appendix 3** | MOOSE checklist |
| **Appendix 4** | Literature search strategy |
| **Appendix 5** | Risk conversion method |
| **Appendix 6** | Baseline participant characteristics according to VTE development |
| **Appendix 7** | Hazard ratios for venous thromboembolism, by quartiles of baseline levels of C-reactive protein in primary analysis |
| **Appendix 8** | Flow of studies included in pooled analysis |
| **Appendix 9** | Characteristics of prospective studies included in meta-analysis |
| **Appendix 10** | Assessment of small study effects by funnel plot and Egger’s test |

**Appendix 1:** STROBE Statement

| **Section/Topic** | Item # | Recommendation | Reported on page # |
| --- | --- | --- | --- |
| **Title and abstract** | 1 | (*a*) Indicate the study’s design with a commonly used term in the title or the abstract | Page 1 |
|  |  | (*b*) Provide in the abstract an informative and balanced summary of what was done and what was found | Page 2 |
| Introduction | | |  |
| Background/rationale | 2 | Explain the scientific background and rationale for the investigation being reported | Page 3-4 |
| Objectives | 3 | State specific objectives, including any pre-specified hypotheses | Page 3-4 |
| Methods | | |  |
| Study design | 4 | Present key elements of study design early in the paper | Study design and participants |
| Setting | 5 | Describe the setting, locations, and relevant dates, including periods of recruitment, exposure, follow-up, and data collection | Study design and participants |
| Participants | 6 | (*a*) Give the eligibility criteria, and the sources and methods of selection of participants. Describe methods of follow-up | Study design and participants |
|  |  | (*b*) For matched studies, give matching criteria and number of exposed and unexposed | Not applicable |
| Variables | 7 | Clearly define all outcomes, exposures, predictors, potential confounders, and effect modifiers. Give diagnostic criteria, if applicable | Assessment of risk markers |
| Data sources/ measurement | 8* | For each variable of interest, give sources of data and details of methods of assessment (measurement). Describe comparability of assessment methods if there is more than one group | Assessment of risk markers |
| Bias | 9 | Describe any efforts to address potential sources of bias | Statistical analyses |
| Study size | 10 | Explain how the study size was arrived at | Statistical analyses |
| Quantitative variables | 11 | Explain how quantitative variables were handled in the analyses. If applicable, describe which groupings were chosen and why | Statistical analyses |
| Statistical methods | 12 | (*a*) Describe all statistical methods, including those used to control for confounding | Statistical analyses |
|  |  | (*b*) Describe any methods used to examine subgroups and interactions | Statistical analyses |
|  |  | (*c*) Explain how missing data were addressed | Not applicable |
|  |  | (*d*) If applicable, explain how loss to follow-up was addressed | Not applicable |
|  |  | (*e*) Describe any sensitivity analyses | Statistical analyses |
| Results | | |  |
| Participants | 13* | (a) Report numbers of individuals at each stage of study—eg numbers potentially eligible, examined for eligibility, confirmed eligible, included in the study, completing follow-up, and analysed | Study design and participants |
|  |  | (b) Give reasons for non-participation at each stage | Study design and participants |
|  |  | (c) Consider use of a flow diagram | Not applicable |
| Descriptive data | 14* | (a) Give characteristics of study participants (eg demographic, clinical, social) and information on exposures and potential confounders | Results; Tables 1 |
|  |  | (b) Indicate number of participants with missing data for each variable of interest |  |
|  |  | (c) Summarise follow-up time (eg, average and total amount) | Results |
| Outcome data | 15* | Report numbers of outcome events or summary measures over time | Results |
| Main results | 16 | (*a*) Give unadjusted estimates and, if applicable, confounder-adjusted estimates and their precision (eg, 95% confidence interval). Make clear which confounders were adjusted for and why they were included | Results; Table 2 |
|  |  | (*b*) Report category boundaries when continuous variables were categorized | Results; Table 2 |
|  |  | (*c*) If relevant, consider translating estimates of relative risk into absolute risk for a meaningful time period |  |
| Other analyses | 17 | Report other analyses done—eg analyses of subgroups and interactions, and sensitivity analyses | Results; Fig. 1. |
| Discussion |  |  |  |
| Key results | 18 | Summarise key results with reference to study objectives | Discussion - Summary of main findings |
| **Limitations** |  |  |  |
| Interpretation | 20 | Give a cautious overall interpretation of results considering objectives, limitations, multiplicity of analyses, results from similar studies, and other relevant evidence | Discussion |
| Generalisability | 21 | Discuss the generalisability (external validity) of the study results | Discussion |
| Other information |  |  |  |
| Funding | 22 | Give the source of funding and the role of the funders for the present study and, if applicable, for the original study on which the present article is based | Page 13 |

**Appendix 2**: PRISMA check-list

| **Section/topic** | **Item No** | **Checklist item** | **Reported on page No** |
| --- | --- | --- | --- |
| **Title** | | | |
| Title | 1 | Identify the report as a systematic review, meta-analysis, or both | 1 |
| **Abstract** | | | |
| Structured summary | 2 | Provide a structured summary including, as applicable, background, objectives, data sources, study eligibility criteria, participants, interventions, study appraisal and synthesis methods, results, limitations, conclusions and implications of key findings, systematic review registration number | 2 |
| **Introduction** | | | |
| Rationale | 3 | Describe the rationale for the review in the context of what is already known | 4 |
| Objectives | 4 | Provide an explicit statement of questions being addressed with reference to participants, interventions, comparisons, outcomes, and study design (PICOS) | 4 |
| **Methods** | | | |
| Protocol and registration | 5 | Indicate if a review protocol exists, if and where it can be accessed (such as web address), and, if available, provide registration information including registration number | Methods |
| Eligibility criteria | 6 | Specify study characteristics (such as PICOS, length of follow-up) and report characteristics (such as years considered, language, publication status) used as criteria for eligibility, giving rationale | Methods |
| Information sources | 7 | Describe all information sources (such as databases with dates of coverage, contact with study authors to identify additional studies) in the search and date last searched | Methods |
| Search | 8 | Present full electronic search strategy for at least one database, including any limits used, such that it could be repeated | Appendix 4 |
| Study selection | 9 | State the process for selecting studies (that is, screening, eligibility, included in systematic review, and, if applicable, included in the meta-analysis) | Methods |
| Data collection process | 10 | Describe method of data extraction from reports (such as piloted forms, independently, in duplicate) and any processes for obtaining and confirming data from investigators | Methods |
| Data items | 11 | List and define all variables for which data were sought (such as PICOS, funding sources) and any assumptions and simplifications made | Methods |
| Risk of bias in individual studies | 12 | Describe methods used for assessing risk of bias of individual studies (including specification of whether this was done at the study or outcome level), and how this information is to be used in any data synthesis | Methods |
| Summary measures | 13 | State the principal summary measures (such as risk ratio, difference in means). | Methods |
| Synthesis of results | 14 | Describe the methods of handling data and combining results of studies, if done, including measures of consistency (such as I^2^ statistic) for each meta-analysis | Methods |
| Risk of bias across studies | 15 | Specify any assessment of risk of bias that may affect the cumulative evidence (such as publication bias, selective reporting within studies) | Methods |
| Additional analyses | 16 | Describe methods of additional analyses (such as sensitivity or subgroup analyses, meta-regression), if done, indicating which were pre-specified | Methods |
| **Results** | | | |
| Study selection | 17 | Give numbers of studies screened, assessed for eligibility, and included in the review, with reasons for exclusions at each stage, ideally with a flow diagram | Appendix 6 |
| Study characteristics | 18 | For each study, present characteristics for which data were extracted (such as study size, PICOS, follow-up period) and provide the citations | Appendix 7 |
| Risk of bias within studies | 19 | Present data on risk of bias of each study and, if available, any outcome-level assessment (see item 12). | Appendix 7 |
| Results of individual studies | 20 | For all outcomes considered (benefits or harms), present for each study (a) simple summary data for each intervention group and (b) effect estimates and confidence intervals, ideally with a forest plot | Figure 2 |
| Synthesis of results | 21 | Present results of each meta-analysis done, including confidence intervals and measures of consistency | Results and Figure 2 |
| Risk of bias across studies | 22 | Present results of any assessment of risk of bias across studies (see item 15) | Not applicable |
| Additional analysis | 23 | Give results of additional analyses, if done (such as sensitivity or subgroup analyses, meta-regression) (see item 16) | Not applicable |
| **Discussion** | | | |
| Summary of evidence | 24 | Summarise the main findings including the strength of evidence for each main outcome; consider their relevance to key groups (such as health care providers, users, and policy makers) | Discussion |
| Limitations | 25 | Discuss limitations at study and outcome level (such as risk of bias), and at review level (such as incomplete retrieval of identified research, reporting bias) | Discussion |
| Conclusions | 26 | Provide a general interpretation of the results in the context of other evidence, and implications for future research | Discussion |
| **Funding** | | | |
| Funding | 27 | Describe sources of funding for the systematic review and other support (such as supply of data) and role of funders for the systematic review | None |

**Appendix 3**: MOOSE checklist

| **Criteria** | | **Brief description of how the criteria were handled in the review** |
| --- | --- | --- |
| **Reporting of background** | |  |
| √ | Problem definition | Elevated baseline circulating C-reactive protein (CRP) has been suggested to the linked to the development of venous thromboembolism (VTE), but the nature and magnitude of the association is uncertain |
| √ | Hypothesis statement | There is an independent association between CRP and VTE risk. |
| √ | Description of study outcomes | VTE |
| √ | Type of exposure | Blood levels of CRP |
| √ | Type of study designs used | Prospective (cohort, case-cohort or “nested case control”) population-based studies |
| √ | Study population | Approximately general populations with no prevalent VTE at baseline |
| **Reporting of search strategy should include** | |  |
| √ | Qualifications of searchers | Setor Kunutsor, MD PhD; Samuel Seidu, MD |
| √ | Search strategy, including time period included in the synthesis and keywords | Time period: from inception of MEDLINE, EMBASE, Web of Science to 10 January, 2017.  **Search strategy:**  In Appendix 4. |
| √ | Databases and registries searched | MEDLINE, EMBASE, and Web of Science |
| √ | Search software used, name and version, including special features | Ovid was used to search EMBASE  Endnote used to manage references |
| √ | Use of hand searching | We searched bibliographies of retrieved papers |
| √ | List of citations located and those excluded, including justifications | Details of the literature search process are outlined in the flow chart. The citation list for excluded studies is available upon request. |
| √ | Method of addressing articles published in languages other than English | We placed no restrictions on language |
| √ | Method of handling abstracts and unpublished studies | None found |
| √ | Description of any contact with authors | Not applicable |
| **Reporting of methods should include** | |  |
| √ | Description of relevance or appropriateness of studies assembled for assessing the hypothesis to be tested | Detailed inclusion and exclusion criteria are described in the Methods section. |
| √ | Rationale for the selection and coding of data | Data extracted from each of the studies were relevant to the population characteristics, study design, exposure, outcome, and possible effect modifiers of the association. |
| √ | Assessment of confounding | We assessed confounding by ranking individual studies on the basis of different adjustment levels, and performed sub-group analyses to evaluate differences in the overall estimates according to levels of adjustment. |
| √ | Assessment of study quality, including blinding of quality assessors; stratification or regression on possible predictors of study results | Study quality was assessed based on the nine-star Newcastle–Ottawa Scale using pre-defined criteria namely: population representativeness, comparability (adjustment of confounders), ascertainment of outcome. Sensitivity analyses by several quality indicators such as study size, duration of follow-up, and adjustment factors. |
| √ | Assessment of heterogeneity | Heterogeneity of the studies was explored with I^2^ statistic that provides the relative amount of variance of the summary effect due to the between-study heterogeneity. |
| √ | Description of statistical methods in sufficient detail to be replicated | Description of methods of meta-analyses, sensitivity analyses, meta-regression and assessment of publication bias are detailed in the methods. We performed random effects meta-analysis with Stata 14. |
| √ | Provision of appropriate tables and graphics | Appendices 6 and 7 |
| **Reporting of results should include** | |  |
| √ | Graph summarizing individual study estimates and overall estimate | Figure 2 |
| √ | Table giving descriptive information for each study included | Appendix 7 |
| √ | Results of sensitivity testing | Sensitivity analysis was conducted to assess the influence of each individual study by omitting one study at a time and calculating a pooled estimate for the remainder of the studies. Results section |
| √ | Indication of statistical uncertainty of findings | 95% confidence intervals were presented with all summary estimates, I^2^ values and results of sensitivity analyses |
| **Reporting of discussion should include** | |  |
| √ | Quantitative assessment of bias | There was no evidence of heterogeneity or publication bias between contributing studies. |
| √ | Justification for exclusion | All studies were excluded based on the pre-defined inclusion criteria in methods section. |
| √ | Assessment of quality of included studies | Brief discussion included in ‘Methods’ section |
| **Reporting of conclusions should include** | |  |
| √ | Consideration of alternative explanations for observed results | Discussed in the context of the results. |
| √ | Generalization of the conclusions | Discussed in the context of the results. |
| √ | Guidelines for future research | Assessment of the potential utility of CRP in the prevention of VTE |
| √ | Disclosure of funding source | No separate funding was necessary for the undertaking of this systematic review. |

**Appendix 4:** Literature search strategy

| Relevant studies, published before 06 January, 2017 (date last searched), were identified through electronic searches not limited to the English language using MEDLINE, EMBASE, and the Science Citation Index databases. Electronic searches were supplemented by scanning reference lists of articles identified for all relevant studies (including review articles), by hand searching of relevant journals and by correspondence with study investigators. The computer-based searches combined search terms related to C-reactive protein and venous thromboembolism without language restriction.  (i) MEDLINE strategy to identify relevant exposures:  (“C-reactive Protein”[MeSH] OR "C-reactive protein"[All Fields])  (ii) MEDLINE strategy to identify relevant outcomes:  ("Venous Thromboembolism"[MeSH] OR “venous thromboembolism”[All Fields] OR "Deep Vein Thrombosis"[MeSH] OR “deep vein thrombosis”[All Fields] OR "Pulmonary Embolism"[MeSH] OR “pulmonary embolism”[All Fields])  (iii) MEDLINE strategy to identify relevant population:  ("humans"[MeSH Terms])  Parts i, ii and iii were combined using ‘AND’ to search MEDLINE. Each part was specifically translated for searching alternative databases. |
| --- |

**Appendix 5:** Risk conversion method

To enable a consistent approach to the meta-analysis and enhance interpretation of findings, relative risk estimates for association of C-reactive protein (CRP) and venous thromboembolism (VTE) that were often differently reported by each study (e.g. per unit change, quintiles, quartiles, thirds, or other groupings) were transformed to consistently correspond to per 1 standard deviation (SD) change in logarithmically transformed baseline levels of CRP using standard statistical methods.^1,2^ Briefly, assuming a normally distributed exposure (e.g. log_e_ CRP) with a log-linear association with VTE risk (i.e. linear relationship between log relative risk estimates and levels of the exposure), conversion factors to convert log relative risks from reported scale comparisons are derived based on the ratio of expected differences in mean levels of the standardised exposure (i.e. SD scale), for the target comparison versus reported comparison. For example, the expected difference in means of the top versus bottom thirds of the standard normal distribution is 2.18 SDs, 2.54 SDs for the top versus bottom quartile, and 2.80 SDs for the top versus bottom quintile. Hence, relative risk estimates reported for top versus bottom thirds comparison can be converted to per 1 SD change by applying a division conversion factor of 2.18 to the log relative risk and its standard error. Similarly, estimates for comparisons of extreme quarters are divided by 2.54 and those for extreme quintiles are divided by 2.80. Conversely, relative risk estimates reported for comparisons of extreme tertiles can be converted to comparisons of extreme quartiles by applying a multiplication conversion factor of 2.54/2.18 to the log relative risk and its standard error and estimates reported for comparisons of extreme quintiles can be converted to comparisons of extreme quartiles by applying a multiplication conversion factor of 2.54/2.80 to the estimates. Similarly, estimates reported per 1 SD can be multiplied by 2.54 to obtain the top versus bottom quartile comparison, and those reported per unit change can be multiplied by 2.54*SD of exposure, to obtain similar comparison. Conversion factors for other possible reported comparisons are derived similarly. The method has been generally been implemented in Stata function --riskconv-- available from <http://www.phpc.cam.ac.uk/ceu/research/erfc/stata/>^3^ and has been used in previous numerous published meta-analyses.^4-6^

| 1. Chêne, G and Thompson, SG. Methods for Summarizing the Risk Associations of Quantitative Variables in Epidemiologic Studies in a Consistent Form, American Journal of Epidemiology, 1996;144:610-621 |
| --- |
| 1. Greenland, S and Longnecker, MP. Methods for trend estimation from summarized dose-response data, with applications to meta-analysis, American Journal of Epidemiology, 1992;135:1301-1309. |
| 1. The Emerging Risk Factors Collaboration. ERFC Methods. Accessed at <http://www.phpc.cam.ac.uk/ceu/research/erfc/methods/> on 26 February 2014. |
| 1. Thompson A and Danesh J. Associations between apolipoprotein B, apolipoprotein AI, the apolipoprotein B/AI ratio and coronary heart disease: a literature-based meta-analysis of prospective studies. J Intern Med. 2006;259:481-492 |
| 1. Chowdhury R, Stevens S, Gorman D, Pan A, Warnakula S, Chowdhury S et al. Association between fish consumption, long chain omega 3 fatty acids, and risk of cerebrovascular disease: systematic review and meta-analysis. BMJ. 2012;345:e6698. |
| 1. Kunutsor SK, Apekey TA, Walley J. Liver aminotransferases and risk of incident type 2 diabetes: a systematic review and meta-analysis. Am J Epidemiol. 2013; 178 (2): 159-171 |

**Appendix 6:** Baseline participant characteristics according to VTE development

|  | **Overall (N=2,420)**  **Mean (SD) or n (%)** | **Without VTE (N=2,301)**  **Mean (SD) or n (%)** | **With VTE (N=119)**  **Mean (SD) or %** |
| --- | --- | --- | --- |
| Log_e_ CRP (mg/l) | 0.34 (0.97) | 0.34 (0.98) | 0.40 (0.93) |
|  |  |  |  |
| ***Questionnaire/Prevalent conditions*** |  |  |  |
| Age at survey (years) | 53.2 (5.0) | 53.2 (5.0) | 54.0 (4.3) |
| Alcohol consumption (g/week) | 75.6 (136.5) | 75.9 (138.5) | 69.0 (88.5) |
| History of diabetes | 99 (4.1) | 95 (4.1) | 4 (3.4) |
| Current smokers | 766 (31.7) | 742 (32.3) | 24 (20.2) |
| History of hypertension | 736 (30.4) | 703 (30.6) | 33 (27.7) |
| History of CHD | 620 (25.6) | 588 (25.6) | 32 (26.9) |
| Lipid medication | 16 (0.7) | 15 (0.7) | 1 (0.8) |
|  |  |  |  |
| ***Physical measurements*** |  |  |  |
| BMI (kg/m^2^) | 26.9 (3.6) | 26.9 (3.6) | 27.4 (4.0) |
| SBP (mmHg) | 134 (17) | 134 (17) | 132 (17) |
| DBP (mmHg) | 89 (11) | 89 (11) | 88 (9) |
| Physical activity (kj/day) | 1,546 (1,482) | 1,547 (1,491) | 1,510 (1,288) |
|  |  |  |  |
| ***Lipid markers*** |  |  |  |
| Total cholesterol (mmol/l) | 5.92 (1.09) | 5.91 (1.08) | 5.98 (1.21) |
| HDL-C (mmol/l) | 1.30 (0.30) | 1.30 (0.30) | 1.28 (0.31) |
| Log_e_ triglycerides (mmol/l) | 0.12 (0.51) | 0.12 (0.51) | 0.18 (0.55) |
|  |  |  |  |
| ***Metabolic, renal, and inflammatory markers*** |  |  |  |
| Fasting plasma glucose (mmol/l) | 5.36 (1.28) | 5.36 (1.28) | 5.39 (1.37) |
| Serum creatinine (µmol/1) | 89.6 (20.7) | 89.6 (21.0) | 90.6 (14.8) |
| Log_e_ GGT (U/L) | 3.13 (0.65) | 3.13 (0.65) | 3.12 (0.64) |

BMI, body mass index; CHD, coronary heart disease; CRP, C-reactive protein; DBP, diastolic blood pressure; GGT, gamma-glutamyltransferase HDL-C, high-density lipoprotein cholesterol; SD, standard deviation; SBP, systolic blood pressure; VTE, venous thromboembolism

**Appendix 7.** Hazard ratios for venous thromboembolism, by quartiles of baseline levels of C-reactive protein in primary analysis

**A**, adjusted for age; **B**, adjusted for age, body mass index, systolic blood pressure, history of hypertension, prevalent coronary heart disease, smoking status, history of diabetes, total cholesterol, lipid medication, physical activity, and gamma-glutamyltransferase

**Appendix 8.** Flow of studies included in pooled analysis

**Appendix 9.** Characteristics of prospective studies included in meta-analysis

| **Lead author, publication year [Reference]** | **Name of study/source of participants** | **Location of study** | **Year(s) of baseline survey** | **Baseline age range (years)** | **% male** | **Mean/mediation duration of follow-up (years)** | **Total no. of participants** | **No. of VTE cases** | **Covariates adjusted for** | **Study quality** |
| --- | --- | --- | --- | --- | --- | --- | --- | --- | --- | --- |
|  |  |  |  |  |  |  |  |  |  |  |
| Ridker, 1997 [13] | PHS | USA | 1982-1984 | 40-84 | 100.0 | 8.0 | 644 | 101 | Random treatment assignment, BMI, DM, history of hypertension, and parental history of CHD | 7 |
| Folsom, 2009 [18] | ARIC | USA | 1996-1998 | 63.0* | 44.6 | 8.3 | 10,505 | 221 | Age, sex, HRT use, race, diabetes, BMI, Factor VIII and aPTT | 8 |
| Mahmoodi, 2009 [14] | PREVEND | Netherlands | 1997-1998 | 49.0* | 50.0 | 8.6 | 8,574 | 129 | Age and sex | 6 |
| Zacho, 2010 [16] | CCHS | Denmark | 1991-1994 | 44.3* | 44.0 | 16.0 | 10,388 | 484 | Age, sex, total cholesterol, LDL-C, HDL-C, Triglycerides, BMI, hypertension, DM, smoking, alcohol, statin use, postmenopausal status, HRT, physical activity | 8 |
| Quist-Paulsen, 2010 [17] | HUNT 2 | Norway | 1995-1997 | 66.2* | 45.4 | 1.0 | 2,020 | 515 | Age and sex | 6 |
| Hald, 2011 [15] | Tromso | Norway | 1994-1995 | 25-84 | NR | 12.5 | 6,426 | 209 | Age, sex, BMI, DM, smoking | 8 |
| Olson, 2014 [19] | REGARDS | USA | 2003-2007 | ≥ 45 | 45.3 | 4.6 | 30,239 | 268 | Age, sex, race, region, BMI, smoking, hypertension, DM, history of CHD or stroke | 7 |
| Cushman, 2016 [47] | CHS | USA | 1989-1993 | 65-100 | NR | 12.6 | 10,409† | 179 | Age, sex, and race | 7 |
| Current study | KIHD | Finland | 1984-1989 | 42-61 | 100.0 | 25.6 | 2,420 | 119 | Age, BMI, SBP, history of hypertension, prevalent CHD, smoking status, DM, total cholesterol, lipid medication, physical activity, and GGT | 8 |
| **Total** |  |  |  |  |  |  | 81,625 | 2,225 |  |  |

*, mean age; †, approximate number of participants; ARIC, Atherosclerosis Risk in Communities; aPTT, activated partial thromboplastin time; BMI, body mass index; CHD, coronary heart disease; CCHS, Copenhagen City Heart Study; DM, diabetes mellitus; GGT, gamma-glutamyltransferase; HDL-C, high-density lipoprotein cholesterol; HRT, hormone replacement therapy; HUNT 2, Nord-Trøndelag Health Study; KIHD, Kuopio Ischemic Heart Disease; LDL-C, low-density lipoprotein cholesterol; NR, not reported; PHS, Physicians’ Health Study; PREVEND, Prevention of Renal and Vascular End-stage Disease; REGARDS, REasons for Geographic And Racial Differences in Stroke; SBP, systolic blood pressure

**Appendix 10.** Assessment of small study effects by funnel plot and Egger’s test

The dotted lines show 95% confidence intervals around the overall summary estimate calculated using a fixed effect model; *P*-value for bias calculated using Egger’s test was 0.743
